# Is it a matter of urgency? A survey of assessments by walk-in patients and doctors of the urgency level of their encounters at a general emergency outpatient clinic in Oslo, Norway

**DOI:** 10.1186/s12873-016-0086-1

**Published:** 2016-07-04

**Authors:** Sven Eirik Ruud, Per Hjortdahl, Bård Natvig

**Affiliations:** Department of General Practice, Institute of Health and Society, University of Oslo, Oslo, Norway; Department of Emergency General Practice, City of Oslo Health Agency, Oslo, Norway

**Keywords:** Emergency medicine, Primary care, General practitioner, Immigrants, Level of urgency, Health literacy

## Abstract

**Background:**

Emergency room (ER) use is increasing in several countries. Variability in the proportion of non-urgent ER visits was found to range from 5 to 90 % (median 32 %). Non-urgent emergency visits are considered an inappropriate and inefficient use of the health-care system because they may lead to higher expenses, crowding, treatment delays, and loss of continuity of health care provided by a general practitioner. Urgency levels of doctor–walk-in patient encounters were assessed based on their region of origin in a diverse Norwegian population.

**Methods:**

An anonymous, multilingual questionnaire was distributed to all walk-in patients at a general emergency outpatient clinic in Oslo during two weeks in September 2009. We analysed demographic data, patient–doctor assessments of the level of urgency, and the results of the consultation. We used descriptive statistics to obtain frequencies with 95 % confidence interval (CI) for assessed levels of urgency and outcomes. Concordance between the patients’ and doctors’ assessments was analysed using a Kendall tau-b test. We used binary logistic regression modelling to quantify associations of explanatory variables and outcomes according to urgency level assessments.

**Results:**

The analysis included 1821 walk-in patients. Twenty-four per cent of the patients considered their emergency consultation to be non-urgent, while the doctors considered 64 % of encounters to be non-urgent. The concordance between the assessments by the patient and by their doctor was positive but low, with a Kendall tau-b coefficient of 0.202 (*p* < 0.001). Adjusted logistic regression analysis showed that patients from Eastern Europe (odds ratio (OR) = 3.04; 95 % CI 1.60–5.78), Asia and Turkey (OR = 4.08; 95 % CI 2.43–6.84), and Africa (OR = 8.47; 95 % CI 3.87–18.5) reported significantly higher urgency levels compared with Norwegians. The doctors reported no significant difference in assessment of urgency based on the patient’s region of origin, except for Africans (OR = 0.64; 95 % CI 0.43–0.96).

**Conclusion:**

This study reveals discrepancies between assessments by walk-in patients and doctors of the urgency level of their encounters at a general emergency clinic. The patients’ self-assessed perception of the urgency level was related to their region of origin.

**Electronic supplementary material:**

The online version of this article (doi:10.1186/s12873-016-0086-1) contains supplementary material, which is available to authorized users.

## Background

Emergency room (ER) use is increasing in several countries [1, 2]. An important factor contributing to the increased use is that of consultations for non-urgent medical problems that could have been handled more appropriately by ordinary primary health-care services [3]. An international literature review reports considerable variability in the proportion of non-urgent ER visits with values ranging from 5 to 90 %, with a median of 32 % [4]. Non-urgent ER consultations are considered an inappropriate and inefficient use of the health-care system because they may lead to higher expenses, crowding, and treatment delays for severely ill patients [2, 5]. Studies report that immigrants tend to use ERs and out-of-hours services for non-urgent reasons [6–9]. Not only do patients using the emergency health-care facilities for non-urgent medical problems create a burden on the emergency health-care services, they may also lose the continuity of health care provided by a regular general practitioner (RGP) [10–12].

In most rural parts of Norway, RGPs handle the primary emergency care needs of patients during the daytime and participate in out-of-hours emergency primary health-care services. In Oslo, patients may find it convenient to use the general emergency clinic, which is part of the larger Oslo Accident and Emergency Outpatient Clinic (OAEOC), and easily accessed 24 h a day, seven days a week. The OAEOC is divided into a general emergency clinic and a trauma clinic, and acts as a gatekeeper to secondary care through a process of referral.

Health status and socio-economic status are important factors influencing the rates of ER use by patients with non-urgent reasons for consultations [13]. Adults and caregivers may seek ER care more often for mild acute illnesses considered as non-urgent because of poor health literacy skills [14, 15]. Cultural differences according to health understanding, poor knowledge about the health-care system, and an inability to make appointments by telephone because of limited language skills, constitute barriers to accessing primary care [7, 16]. Illegal immigrant status may contribute to the increased use of ER services. In Norway, citizens who are registered in the National Population Register and asylum seekers including their families are entitled to register with a RGP [17]. Most immigrants in Oslo are registered or asylum seekers and they have a legal right to choose to attend either their RGP during office hour or the emergency clinic when in need for an immediate consultation. A RGP is a general practitioner who has entered into an agreement with the local authorities to act as a primary health-care provider for those citizens who are registered on their list. Undocumented immigrants, rejected asylum seekers, and short-term labour immigrants fall outside the RGP system, but they have the right to receive emergency health care. For them the emergency clinic may be the only relevant source of health care service to attend.

The purpose of the study was to provide data about how patients and doctors assess the urgency level of the emergency encounter to better understand the reasons for emergency clinic utilization. This knowledge may provide potential useful policy implications in way of developing educational intervention programs to increase health literacy and to secure equity health care service for specific vulnerable groups. The primary aim of this study was to explore how patients and their doctors perceived the level of urgency for obtaining medical assistance and to determine the concordance between their assessments in the diverse population of walk-in patients attending a general emergency outpatient clinic in Oslo, Norway. The secondary aim was to explore whether there were any differences in the assessments of the level of urgency by Norwegians, immigrants, and subgroups of immigrants based on their region of origin. Finally, we explored whether there were any associations between the level of urgency for the consultation as perceived by patients and the result of the consultation.

## Methods

### Setting and study design

The present study is based on data obtained from a survey conducted by means of a questionnaire distributed to walk-in patients at the general emergency clinic at the OAEOC during two weeks in September 2009. The general emergency clinic is operated by the Municipality of Oslo. In 2009, the clinic handled approximately 80,000 emergency contacts. Immigrants and Norwegian-born citizens with immigrant parents comprised 42 % of the emergency walk-in contacts [18].

The general emergency clinic directly handles patients in need of emergency health care, without referrals. Patients arrive either alone or together with their relatives (walk-in patients), or are brought in by emergency services (ambulance, police, and emergency outreach teams). At the clinic, the walk-in patients are seen by a specialist nurse for registration and triage before waiting for their turn to be seen by a doctor. Patients brought in by emergency services enter the general emergency clinic via a separate entrance, and they are treated according to the level of urgency of their condition.

Patients were registered for the study on a 24-h basis. After triage, all walk-in patients were invited to participate in the study. They were then asked to answer a 15-item questionnaire while in the waiting room. Patients not able to sit in the waiting room were offered a bed in an examination room were they filled in the questionnaire, either themselves or together with a relative or guardian. The questionnaire included items related to their country of birth, age, gender, the countries of their parents’ birth, and their assessment of the urgency level for their visit (see Additional file 1). Children younger than 16 years and elderly patients were assisted by relatives or on-site health-care personnel when answering the questions. The questionnaire consisted of two parts: one for the patient and one for the doctor. The patients returned their completed part of the questionnaire to the doctor, who completed the appropriate part at the end of the consultation. The doctors registered the time of day and their objective assessment of the level of urgency for the consultation.

To accommodate the multiple nationalities of the patients, the questionnaire and attached information sheets were available in seven languages: Norwegian, English, Polish, Somali, Sorani (Kurdish), Farsi (Persian), and Urdu. The Municipal Interpreting and Translation Service of Oslo was consulted regarding the languages selected, and prepared the translations of the original questionnaire. Each language edition was examined and proofread by an independent translator, who then compared it to the original text in Norwegian. Inconsistencies were resolved in consultation with the translators.

### Inclusion

Walk-in patients of all ages except patients attending scheduled return visits were included. Patients brought in by emergency services or who were intoxicated or having an acute psychiatric episode were considered not eligible for inclusion. The included patients were categorized by their immigration status and country of birth, according to the criteria and the definitions provided by Statistics Norway [19]. Patients were defined as being of non-Norwegian origin if they and both of their parents were born abroad (first-generation immigrants) or if they were born in Norway, but both parents were born abroad (second-generation immigrants). Other constellations were classified as Norwegians. Patients were divided into groups of region of origin based on their birth country, or their mother’s country of birth if the patient was born in Norway.

### Analyses

The patient and the doctor categorized the urgency level related to their encounter according to three pre-defined levels. I: ‘very urgent. I/(The patient) must have help within an hour or sooner’, II: ‘fairly urgent. I/(The patient) must have help within a few hours’, and III: ‘Not so urgent. I/(The patient) could perhaps have waited until tomorrow’. Descriptive statistics and a Z-proportion test were used to obtain frequencies with 95 % confidence intervals for nominal and ordinal categorical variables. To explore the difference in how patients perceived the level of urgency in light of the doctors’ overall evaluation, we estimated the agreement (concordance) between their assessments using a Kendall tau-b correlation coefficient. We used binary logistic regression modelling to quantify associations of explanatory variables and outcomes according to the urgency level assessments. The dependent variable assessments by both patients and doctors was dichotomized into ‘immediate’ (categories I and II) and ‘non-urgent’ (category III). The independent variable was region of origin, adjusted for gender, age, self-reported RGP status, and time of consultation. Data were analysed using IBM SPSS Statistics for Windows (version 22.0) and Stata (version 13.3). Statistical significance was set at 5 % (*p* < 0.05).

## Results

**Fig. 1:**
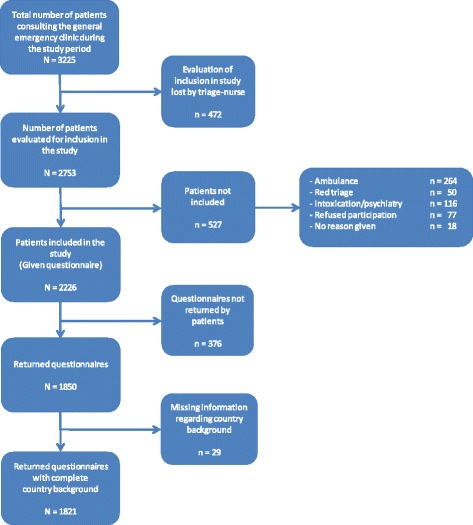
Flow chart of participant inclusion in the study

Of the 3225 patients who attended the general emergency clinic during the registration period, 525 were admitted by emergency services (ambulance, police, and emergency outreach teams), and therefore not included as walk-in patients. Because of practical constraints such as crowding and time limitations at the emergency department, 472 (15 %) were lost to evaluation for inclusion by triage nurses (Fig. 1). Of the 2226 patients included, 1821 (82 %) returned a complete questionnaire that included their country background; 376 left before consultation with the doctor probably because of long waiting times (sometimes 2–6 h), or forgot to hand in the questionnaire during the consultation. Due to missed information regarding the patient’s country of origin, 29 were rejected from the data-analysis. Immigrants constituted 42 % of the study sample (Table 1). Patients with an immigrant background represented 71 nationalities. Among those, 78 % preferred the Norwegian language version of the questionnaire, 11 % the English version, 5 % Polish, 4 % Somali, 1 % Urdu, 1 % Farsi (Persian), and 0.3 % Sorani (Kurdish). Fifty-eight per cent of the Norwegian patients were female and 51 % of the immigrants were female. The mean age of the patients was 29.1 years for Norwegians and 26.5 years for immigrants (Table 1). There was a significant difference in the proportion of patients who reported being registered with the RGP scheme between Norwegians (96 %) and immigrants (77 %). Approximately 50 % of the patients attended the emergency outpatient clinic during normal office hours (08:00 a.m. – 03:59 p.m.). There was no significant difference in the time of consultation between Norwegians and immigrants.

**Table 1:** Characteristics of the patients at the general emergency clinic (*n* = 1821)

	Norwegians			Immigrants^a^		
	*n*	%	95 % CI	*n*	%	95 % CI
Number of patients	1053	57.8	(55.5–60.0)	768	42.2	(39.4–43.9)
Region of origin (immigrants^a^)						
Nordic countries				131	7.2	(6.1–8.5)
Western Europe, North America, and Oceania				51	2.8	(2.1–3.7)
Eastern Europe				121	6.6	(5.6–7.9)
Asia including Turkey				259	14.2	12.7–15.9)
Africa				179	9.8	(8.5–11.3)
Latin America				27	1.5	(1.0–2.2)
Gender						
Female	609	58.3	(55.3–61.3)	386	51.2	(47.6–54.8)
Male	435	41.7	(38.7–44.7)	368	48.8	(45.3–52.4)
Mean age, years (min–max)	29.1 (0–88)			26.5 (0–82)		
Self-reported RGP status						
Registered	1008	95.7	(94.3–96.8)	578	76.6	(73.4–79.4)
Not registered	32	3.0	(2.2–4.3)	165	21.9	(19.1–24.9)
Do not know	13	1.2	(0.7–2.1)	12	1.6	(0.9–2.8)
Time of consultation						
08:00 a.m. – 03:59 p.m.	443	50.1	(46.8–53.4)	345	51.7	(47.9–55.5)
04:00 p.m. – 10:59 p.m.	346	39.1	(36.0–42.4)	272	40.8	(37.1–44.6)
11:00 p.m. – 07:59 a.m.	95	10.7	(8.9–13.0)	50	7.5	(5.7–9.8)

^a^Including both first- and second-generation immigrants

**Fig. 2:**
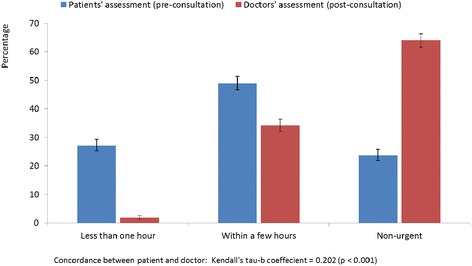
Assessments of how patients and doctors estimate the level of urgency for their consultation (95 % CI)

Figure 2 shows the assessments by patients and their doctors of the urgency level for their consultation. The perception of urgency levels by patients were subjective assessments experienced on admission (pre-consultation), while the assessments by doctors were objectively based on information at discharge (post-consultation). Twenty-seven per cent of patients considered that they needed attention within an hour, while 2 % of the doctors evaluated the level of urgency similarly. Twenty-four per cent of patients considered the reason for the consultation to be non-urgent, while the doctors considered 64 % of the walk-in patients to be presenting non-urgent health-care enquiries. The concordance between the assessments by the patients and their doctors was in general positive, but low, with a Kendall tau-b coefficient = 0.202 (*p* < 0.001).

Table 2 shows the assessments by patients and doctors of the urgency level, and the concordance between their assessments. The proportion of patients perceiving the urgency level as the need to obtain assistance within ‘less than one hour’ was highest among Africans (55 %), Eastern Europeans (50 %), and those from Asia and Turkey (46 %). Among Norwegians and patients from the Nordic countries, the proportion of patients assessing a high level of urgency was lower, at 18 and 16 %, respectively. Almost 40 % of the Nordic patients evaluated their level of urgency as non-urgent. The agreement between the assessment by patients and doctors of the level of urgency for health care was in general positive, but low. The highest concordance was found for Norwegians with a Kendall tau-b coefficient = 0.296 (*p* < 0.001). Sub analysis of the concordance of assessments for consultation results found a Kendall tau-b score = 0.143 (*p* < 0.001) for patients whom received their treatment on site, 0.145 (*p* = 0.029) for patients admitted to hospital/decision unit or referred to specialist, and 0.185 (*p* = 0.008) for those referred for follow-up by their RGP (see Additional file 2).

**Table 2:** Patients’ and doctors’ assessments of urgency level for the consultation and agreement between their assessments

			Nordic countries	Western Europe, North America, Oceania	Eastern Europe	Asia including Turkey	Africa	Latin America
	Norwegians	Immigrants^a^						
	*n* = 867	*n* = 620	*n* = 118	*n* = 38	*n* = 101	*n* = 196	*n* = 145	*n* = 22
	%	%	%	%	%	%	%	%
Assessment by patients of urgency level								
Less than one hour	17.5	40.2	16.1	23.7	48.5	43.4	54.5	36.4
Within a few hours	54.0	43.2	44.1	47.4	39.6	46.9	38.6	45.5
Non-urgent	28.5	16.6	39.8	28.9	11.9	9.7	6.9	18.2
Assessment by doctors of urgency level								
Less than one hour	1.6	1.9	0.8	5.3	2.0	2.0	1.4	4.5
Within a few hours	36.8	29.7	28.0	31.6	29.7	35.7	21.4	36.4
Non-urgent	61.6	68.4	71.2	63.2	68.3	62.2	77.2	59.1
Agreement using a Kendall tau-b coefficient	0.296**	0.129**	0.222*	−0.120	0.127	0.195*	0.090	0.196

^a^Including both first- and second-generation immigrants, **p < 0.05, ** *p* < 0.001

Missing pair of observations: Total; *n* = 334 (18.3 %)

Table 3 shows the results of the binary logistic regression analysis of patients’ and doctors’ assessments of the urgency level, both unadjusted and adjusted for gender, age, self-reported RGP status, and time of consultation. Adjusted analysis showed that patients from Eastern Europe, odds ratio (OR) = 3.04 (95 % CI 1.60–5.78), Asia and Turkey OR = 4.08 (95 % CI 2.43–6.84), and Africa OR = 8.47 (95 % CI 3.87–18.5), all reported a significantly higher perception of the urgency level compared with Norwegians. The doctors reported no significant difference in their assessment of the urgency based on the region of origin of the patients, except for assessing a lower urgency level for Africans with an OR = 0.64 (95 % CI 0.43–0.96) compared to Norwegians. Both patients and doctors reported significantly higher levels of urgency for patients attending the emergency clinic during the night. Assessment by both patients and doctors showed that the age of the patient contributed to the assessment of a significantly higher level of urgency, while gender and RGP registration status did not significantly influence the assessments of urgency. Analysis with a proxy variable of occupational status as an indicator for socioeconomic status, made no significant changes to the associations for assessments of urgency level based on the patients’ region of origin (see Additional file 3).

**Table 3:** Logistic regression analysis of patients’ and doctors’ assessment of urgency level (dependent variable: ‘immediate’ versus ‘non-urgent’)

	Assessment by patients		Assessment by doctors	
	Unadjusted	Adjusted	Unadjusted	Adjusted
	OR (95 % CI)	OR (95 % CI)	OR (95 % CI)	OR (95 % CI)
Country/region of origin				
Norway	1	1	1	1
Nordic countries	0.66 (0.45–0.96)*	0.99 (0.60–1.64)	0.64 (0.42–0.97)*	0.81 (0.50–1.32)
Western Europe/North America and Oceania	1.18 (0.62–2.25)	1.16 (0.55–2.42)	0.92 (0.47–1.81)	1.06 (0.53–2.12)
Eastern Europe	3.18 (1.76–5.74)**	3.04 (1.60–5.78)**	0.76 (0.49–1.16)	0.81 (0.51–1.30)
Asia with Turkey	3.68 (2.34–5.77)**	4.08 (2.43–6.84)**	1.04 (0.77–1.41)	1.01 (0.73–1.39)
Africa	4.25 (2.42–7.47)**	8.47 (3.87–18.5)**	0.49 (0.33–0.72)**	0.64 (0.43–0.96)*
Latin America	1.70 (0.64–4.55)	1.82 (0.59–5.55)	1.21 (0.53–2.80)	1.09 (0.45–2.61)
Gender				
Female		1		1
Male		1.14 (0.87–1.49)		0.95 (0.76–1.19)
Age (years)				
0–19		1		1
20–39		0.91 (0.67–1.24)		1.28 (0.97–1.68)
40–59		2.28 (1.42–3.66)**		2.05 (1.46–2.88)**
≥60		2.73 (1.50–4.97)**		2.46 (1.61–3.76)**
Self-reported RGP status				
Registered		1		1
Not registered		0.68 (0.43–1.07)		0.86 (0.57–1.29)
Time of consultation				
08:00 a.m. – 03:59 p.m.		1		1
04:00 p.m. – 10:59 p.m.		1.03 (0.79–1.35)		1.16 (0.92–1.46)
11:00 p.m. – 07:59 a.m.		2.91 (1.61–5.27)**		2.35 (1.61–3.43)**

*RGP* regular general practitioner

Norwegians used as the reference group. OR (odds ratio)

*Significant result at the *p* < 0.05 level, ** *p* < 0.001

**Fig. 3:**
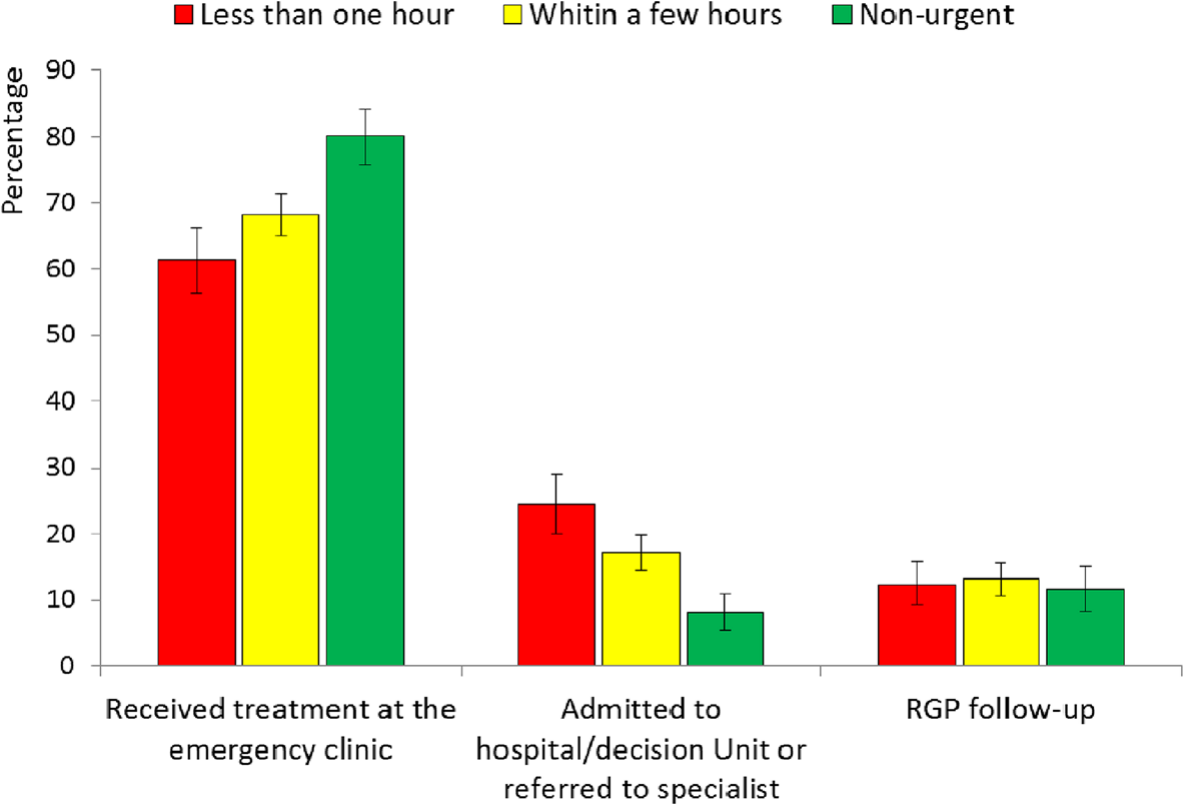
Consultation results based on self-assessed level of urgency by patients

**Fig. 4:**
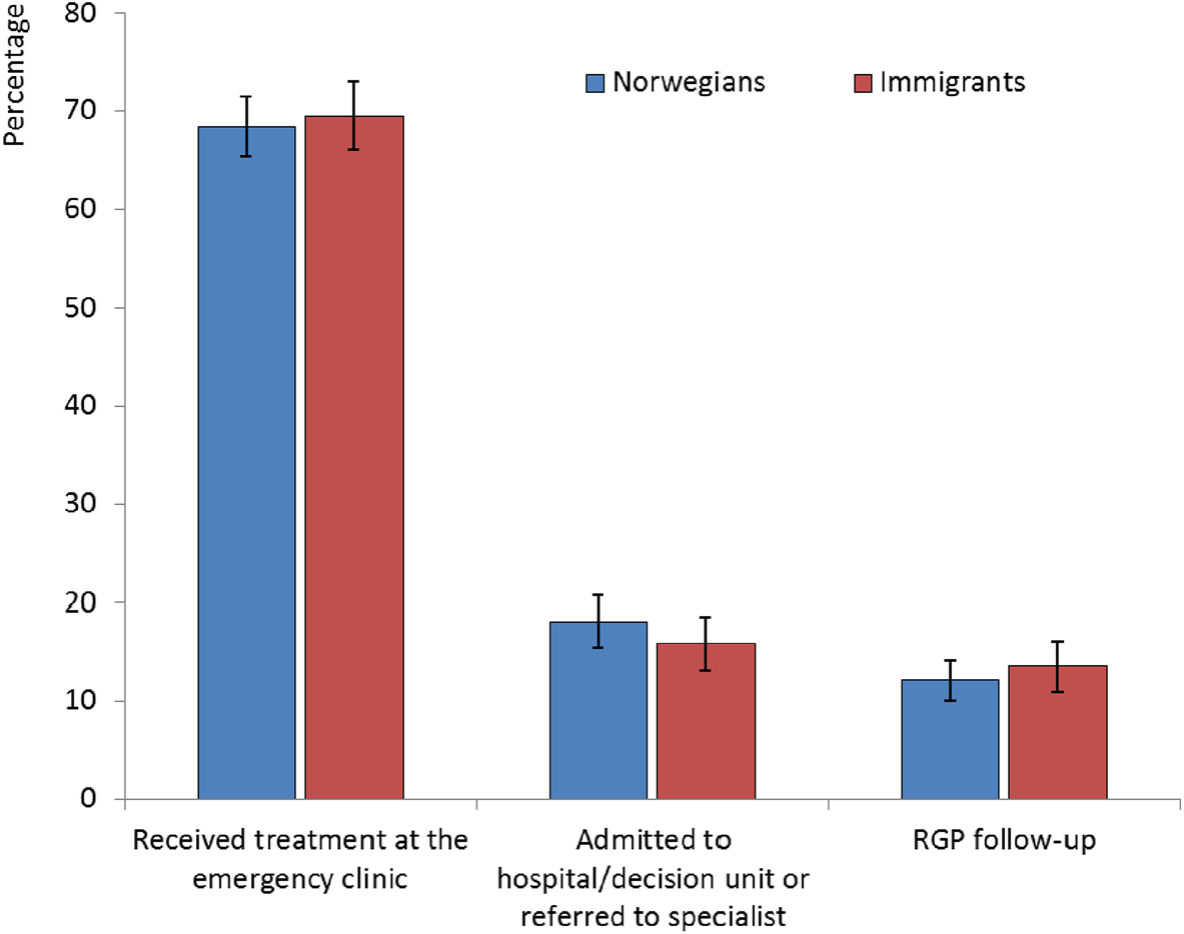
Consultation results for Norwegians and immigrants

The majority of the patients (69 %) received their treatment on site, while 17 % were admitted to the hospital or referred to a specialist, and 13 % were referred for follow-up by their RGP (Fig. 3). In addition, 1 % of both Norwegian and immigrant patients were referred to other institutions: nursing homes, rehabilitation units, and social care units. There was no significant difference in the number of referrals between patients who considered that help was needed in ‘less than one hour’ and those that felt that it was needed ‘within a few hours’. Among patients assessing their level of urgency as non-urgent, a significantly higher proportion of cases were handled on site, and fewer patients were referred to secondary care. There was no significant difference in referrals between Norwegians and immigrants as a group (Fig. 4). Distinguishing the patients according to their region of origin showed no significant differences in referrals compared with Norwegians, except for Africans where a lower proportion were admitted to secondary care: Africans 9 % (95 % CI 6–15), Norwegians 18 % (95 % CI 16–21), *p* < 0.008 (see Additional file 4).

## Discussion

### Findings of the study

The present study demonstrates a discrepancy between assessments of the level of urgency by walk-in patients and doctors for consultations at a general emergency outpatient clinic. Almost two-thirds of the walk-in patients seen at the emergency clinic were assessed by doctors as presenting with a non-urgent medical problem that could have waited for medical attention until next day, while only about one-quarter of the patients shared this assessment of their consultation. Immigrants from Eastern Europe, Asia and Turkey, and Africa more often assessed a significantly higher level of urgency for their consultation compared with Norwegians. In the present study, we distinguished the assessment of the level of urgency based on the region of origin of the patients, which contributes to further knowledge about emergency health-care use and health-seeking behaviour in a diverse population of walk-in patients.

There is no agreed-upon international definition regarding non-urgent emergency health-care visits [4]. In the present study, we defined a non-urgent reason for the consultation as one that could have waited for medical attention until the next day’. Studies have shown a consistent discrepancy in perspectives on urgency between health-care professionals and their patients [20, 21]. Assessments made by health-care professionals are mainly based on urgency of the medical problems, while assessments by patients are based on perceptions of medical factors, feelings (e.g., pain, anxiety), accessibility to health-care resources, and practical concerns surrounding the medical problem. In the present study, the perceptions of the level of urgency by patients were assessments experienced on admission to the emergency clinic (pre-consultation). The assessments by doctors were based on information given in the patient history, by clinical examination, and supplementary diagnostic tests before discharge (post-consultation). This may, at least partly, explain the low concordance between the assessments of level of urgency by patients and doctors in the present study. The patients and physicians may have had a higher degree of concordance if the assessments had been done at the same point in the evaluation. This was difficult to achieve due to preconditioned information received by the doctor before the encounter (i.e., laboratory tests, ECG or reports given by the nurses). Our results emphasize, however, that all groups of walk-in patients, including immigrants, subgroups of immigrants and natives, overestimate their urgency level correlated to the overall evaluation of the doctors. A somewhat surprising finding was that a substantial proportion of patients (11 %) admitted to hospital considered their urgency level to be “non-urgent”, and that the doctors assessed 17 % of the patients admitted to have a “non-urgent” urgency level. An explanation for this finding could be that the emergency clinic in Oslo takes care of many people with low social support, i.e., drug addicted with no permanently place to stay and elderly with insufficient health care support at home. Even though the medical conditions are not urgent they are admitted to a hospital largely due to psychosocial problems.

From the perspective of the patients, they do not necessarily consider their medical problem to be urgent, but at the same time, they urgently wish to have a clarification of their medical problem. For them, in choosing between their RGP or attending an emergency health-care clinic, the general emergency clinic may be the most suitable place and the most efficient provider to fulfil their medical goals. The emergency care facility can deliver a full range of medical services, regardless of the presenting complaint, and it is accessible 24 h a day and seven days a week [22]. These numerous advantages do not exist in RGP offices, where appointment availability can be sparse and opening hours restricted. One study reports that healthy young adults, who were mostly registered with a GP, used emergency services because of convenience and ease of access rather than dissatisfaction with their GP [23]. Lack of permanent registration with the RGP scheme may force patients to use emergency care services for non-urgent medical problems [8, 24]. A study conducted at the OAEOC reported that 96 % of the Norwegians taking part stated that they were registered with a permanent GP versus 77 % in the immigrant population, with lowest registration rates among labour immigrants, particularly those from Sweden and Poland [18].

Immigrants in the form of refugees and asylum seekers share a number of health risks before, during, and after they migrate [25]. They may have different disease profiles from those of the population resident in the host country. Factors contributing to the assessment of a higher level of urgency may include different cultural understandings of health, negative evaluations of their own health status and illness, harmful health effects of perceived prejudice and discrimination (‘minority stress’), and poor health condition in general [26–29]. The way immigrants navigate in a “foreign” land, with a new language, new laws and rules that are unfamiliar, as well as a new health care system, is important for acknowledging the reasons behind their assessments of urgency. Health status and socio-economic status are also important factors influencing the use of emergency services by patients with non-urgent requirements [13]. Adults and caregivers may seek emergency care more often for mild acute illnesses considered non-urgent because of poor health literacy skills [14]. For instance, a medical condition with fever and diarrhoea in an African context may indicate a potentially severe disease such as malaria or dysentery, but in Norway, these symptoms are more commonly caused by a relatively harmless viral gastroenteritis. Low health literacy has been associated with decreased use of preventive services, higher use of acute health-care services, poor health status, and worse health outcomes, including increased hospitalization rate and mortality [15].

### Findings related to previous research

An international literature review shows considerable variability in the proportion of non-urgent ER visits, ranging from 5 to 90 %, with a median of 32 % [4]. Another review reveals that the prevalence of inappropriate ER use varied from 20 to 40 % and was associated with age and income [3]. In the present study, doctors assessed 64 % of the walk-in patients to have non-urgent reasons for their consultation at an emergency outpatient clinic. Durand et al. [4] state that selection bias seems to occur in urgency studies because of the number of patients excluded. Authors have systematically excluded patients requiring immediate treatment and those with communication difficulties, resulting in a higher proportion of non-urgent ER visits than if calculated on the entire patient population visiting the ER. If we consider the patients in our study arriving by emergency services (ambulance, police, and emergency outreach teams) to have an appropriate and urgent health-care enquiry, the proportion of non-urgent enquiries is reduced to approximately 40 % for the entire patient population at the general emergency clinic. In the present study, 27 % of all patients assessed their need for help as being needed within ‘less than one hour’, varying from 18 % among Norwegians, 16–24 % of Western origin (Nordic countries, Western Europe, North America, and Oceania), 49 % of Eastern Europeans, and 36–55 % of patients with non-Western origin (Asia including Turkey, and Africa and Latin America). The same trend is reported in a study from an ER in Copenhagen, where patients of Danish origin (24 %), Western origin (27 %), Middle Eastern regions (63 %), and other non-Western origin (52 %) responded that they needed acute help (<1 h) [7]. An important finding of the present study is the low concordance of assessment of the level of urgency between patients and doctors. A study from a rural Australian Emergency Department found no correlation between patient perception of urgency and triage category [30]. In Saudi Arabia, approximately two-thirds (65.3 %) of Canadian Triage and Acuity Scale (CTAS) V patients and one-third (31.8 %) of CTAS IV patients believed their condition was more urgent than their triage nurse rating [31]. To our knowledge, there are no other studies analysing differences in concordance of assessments of level of urgency by walk-in patients and their doctors between various immigrant groups. However, a study from Italy reports that the consistency of level of urgency and priority made by nurses at entry and exit triage made by physicians was similar for all citizenship groups, with a Kendall tau coefficient of between 0.78 and 0.88 [32].

### Strengths and limitations

To our knowledge, no other quantitative studies have analysed the concordance between the assessment of the urgency level for consultations by walk-in patients and by doctors at an emergency clinic. However, a semi-structured interview study has highlighted discrepancies between the perceptions of ER patients and those of health-care professionals [21]. The response rate in our study (82 %) was high compared with similar studies [7, 24]. However, 472 (15 %) of the patients were lost for inclusion and registration by the triage nurses. To our knowledge, these patients were mostly emergency admissions brought in by emergency services, which were not included in any case. Because the aim of the present survey was to evaluate assessments of urgency by walk-in patients, we assumed that the included participants are representative of the entire patient population attending the general emergency outpatient clinic. The 376 persons who left before consultation may have been different from those who completed the survey. Probably these patients considered their urgency level less urgent since they decided to leave the emergency clinic before an examination by the doctor, or they might have managed to make an appointment with their RGP during the waiting time. This might introduce a bias in the distribution of urgency levels in our study in favour of more patients assessing the urgency level to be high.

Our data may seem a little outdated since the survey was conducted back in 2009. There have, however, not been any major changes in health care organization during this period. The proportion of immigrants resident in Oslo has increased from 27 to 33 % from 2009 to 2016, but we do not think this will have any major impact on the results in this study. A limitation of the study is the lack of good data for socioeconomic status such as educational level and household income. However, the model was analysed using occupational status as a proxy variable and indicator for socioeconomic status. This model made no significant changes to the associations for assessments of urgency level based on the patients’ region of origin (see Additional file 3). Another limitation applies to the lack of a measure of co-morbidity. The level of co-morbidity could be relevant in interpreting the difference between the doctor`s and patient’s assessment of urgency in the model. We decided to include both first- and second-generation immigrants as one group in our analyses. As a result, we may have overlooked important differences between these two categories. However, because many second-generation immigrants are minors, the questionnaire was completed by their accompanying caregiver, and thus reflects the attitudes and perceived level of urgency on the part of the caregiver [18]. It is possible that less-integrated immigrants were more unlikely to answer the questionnaire because of language barriers and illiteracy. Patients for whom a translated questionnaire was not available may have been excluded from the study. Nevertheless, patients presenting to the emergency clinic often arrive with a friend or family member to interpret for them, reflected by the high proportion of use of the Norwegian language version. A limitation of the study is that we were not able to evaluate differences in urgency assessments on country background because of the sample size. Immigrants from different countries in Africa and Asia are diverse, and cultural differences that we were unable to address will exist within these regions.

### Relevance of the findings and suggestion for further research

Our findings have implications for the organization of the primary health-care system in Norway. The consequences of increased utilization of emergency services by patients with non-urgent health-care enquiries decrease access for patients with genuine emergency cases, reduce the quality of care (prolonged waiting times, delayed diagnoses and treatments, delayed care of seriously ill patients), and lead to higher expenses for the health-care system [2, 3, 5, 21]. To establish continuity in health care, it is important that patients attend their RGP for non-urgent health problems. Thus, general initiatives should be taken to improve access to primary health-care services run by RGPs and to enable appointments to be made at short notice. Further initiatives must be taken to establish supplementary primary health-care centres for immigrants whom do not qualify for registration with the RGP scheme or to develop a system where each RGP is required to see a certain number of persons who would not otherwise qualify. Improving the health literacy skills in the population in general can potentially affect health-care-seeking behaviour and reduce non-urgent reasons for visits to emergency clinics. An interesting finding of the present study is the different assessment of the level of urgency between Norwegians and subgroups of immigrants. Further research is needed to explore the possible reasons for this difference.

## Conclusion

This study reveals a discrepancy between how walk-in patients and doctors define the level of urgency of their encounters at a general emergency outpatient clinic. Approximately two-thirds of walk-in consultations were considered by doctors as non-urgent. The self-assessed perception of the level of urgency by patients was related to their region of origin.

## Abbreviations

CI, confidence interval; ER, emergency room; OAEOC, The Oslo Accident and Emergency Outpatient Clinic; OR, odds ratio; RGP, regular general practitioner

## Additional files

Additional file 1: English version of the questionnaire used in the study. The questionnaire has three parts: I. Request and information for participation in the study; II. Fifteen-item questionnaire completed by patients and III. Seven-item registration form for doctors. Part III is in Norwegian. (PDF 209 kb) Additional file 2: Concordance for urgency assessment levels between patients and doctors stratified by consultation results. Additional table showing the concordance for urgency assessment levels between patients and doctors stratified by consultation results. A: Received final treatment at the emergency clinic, B: Admitted to hospital/decision unit or referred to specialist and C: RGP follow up. Additional file 3: Logistic regression analysis for patients’ and doctors’ assessment of urgency level. Additional table presenting a logistic regression model for patients’ and doctors’ assessment of urgency level (dependent variable: ‘immediate’ versus ‘non-urgent’) using occupational status as a proxy for socioeconomic status. (PDF 181 kb) Additional file 4: Consultation results for Norwegians and immigrants based on region of origin. Additional table showing the consultation results according to the patients’ region of origin. (PDF 117 kb)

## References

[1] Rowe BH, Guo X, Villa-Roel C, Schull M, Holroyd B, Bullard M (2011). The role of triage liaison physicians on mitigating overcrowding in emergency departments: a systematic review. Acad Emerg Med.

[2] Hoot NR, Aronsky D (2008). Systematic review of emergency department crowding: causes, effects, and solutions. Ann Emerg Med.

[3] Carret ML, Fassa AC, Domingues MR (2009). Inappropriate use of emergency services: a systematic review of prevalence and associated factors. Cad Saude Publica.

[4] Durand AC, Gentile S, Devictor B, Palazzolo S, Vignally P, Gerbeaux P (2011). ED patients: how nonurgent are they? Systematic review of the emergency medicine literature. Am J Emerg Med.

[5] Moskop JC, Sklar DP, Geiderman JM, Schears RM, Bookman KJ (2009). Emergency department crowding, Part 1—Concept, causes, and moral consequences. Ann Emerg Med.

[6] Norredam M, Krasnik A, Moller Sorensen T, Keiding N, Joost Michaelsen J, Sonne NA (2004). Emergency room utilization in Copenhagen: a comparison of immigrant groups and Danish-born residents. Scand J Pub Health.

[7] Norredam M, Mygind A, Nielsen AS, Bagger J, Krasnik A (2007). Motivation and relevance of emergency room visits among immigrants and patients of Danish origin. Eur J Public Health.

[8] Petersen LA, Burstin HR, O’Neil AC, Orav EJ, Brennan TA (1998). Nonurgent emergency department visits: the effect of having a regular doctor. Med Care.

[9] Ballotari P, D’Angelo S, Bonvicini L, Broccoli S, Caranci N, Candela S (2013). Effects of immigrant status on Emergency Room (ER) utilisation by children under age one: a population-based study in the province of Reggio Emilia (Italy). BMC Health Serv Res.

[10] Huntley A, Lasserson D, Wye L, Morris R, Checkland K, England H (2014). Which features of primary care affect unscheduled secondary care use? A systematic review. BMJ Open.

[11] Starfield B, Shi L, Macinko J (2005). Contribution of primary care to health systems and health. Milbank Q.

[12] Freeman GK, Olesen F, Hjortdahl P (2003). Continuity of care: an essential element of modern general practice?. Fam Pract.

[13] Khan Y, Glazier RH, Moineddin R, Schull MJ (2011). A population-based study of the association between socioeconomic status and emergency department utilization in Ontario, Canada. Acad Emerg Med.

[14] Morrison AK, Schapira MM, Gorelick MH, Hoffmann RG, Brousseau DC (2014). Low caregiver health literacy is associated with higher pediatric emergency department use and nonurgent visits. Acad Pediatr.

[15] Griffey RT, Kennedy SK, McGownan L, Goodman M, Kaphingst KA (2014). Is low health literacy associated with increased emergency department utilization and recidivism?. Acad Emerg Med.

[16] Rue M, Cabre X, Soler-Gonzalez J, Bosch A, Almirall M, Serna MC (2008). Emergency hospital services utilization in lleida (Spain): a cross-sectional study of immigrant and Spanish-born populations. BMC Health Serv Res.

[17] HELFO [The Norwegian Health Economics Administration]. The General Practitioner (GP) scheme. 2015. https://helsenorge.no/foreigners-in-norway/general-practitioner. Accessed 25 May 2015.

[18] Ruud SE, Natvig B, Aga R, Hjortdahl P (2015). Immigrants’ use of emergency care services–a survey among patients attending the Oslo accident and emergency outpatient clinic. BMC Emerg Med.

[19] Befolkning – innvandrere og Norskfødte med innvandrerforeldre [Population –immigrants and Norwegian-born with immigrant parents]. 2013. http://www.ssb.no/befolkning/statistikker/innvbef/aar/2013-04-25?fane=om#content. Accessed 2 Nov 2013.

[20] Sanders J (2000). A review of health professional attitudes and patient perceptions on ‘inappropriate’ accident and emergency attendances. The implications for current minor injury service provision in England and Wales. J Adv Nurs.

[21] Durand AC, Palazzolo S, Tanti-Hardouin N, Gerbeaux P, Sambuc R, Gentile S (2012). Nonurgent patients in emergency departments: rational or irresponsible consumers? Perceptions of professionals and patients. BMC Res Notes.

[22] Richardson LD, Asplin BR, Lowe RA (2002). Emergency department crowding as a health policy issue: past development, future directions. Ann Emerg Med.

[23] Amiel C, Williams B, Ramzan F, Islam S, Ladbrooke T, Majeed A (2014). Reasons for attending an urban urgent care centre with minor illness: a questionnaire study. Emerg Med J.

[24] Hargreaves S, Friedland JS, Gothard P, Saxena S, Millington H, Eliahoo J (2006). Impact on and use of health services by international migrants: questionnaire survey of inner city London A&E attenders. BMC Health Serv Res.

[25] Norredam M, Nielsen SS, Krasnik A (2010). Migrants’ utilization of somatic healthcare services in Europe—a systematic review. Eur J Public Health.

[26] Blom S (2010). Sosiale forskjeller i innvandreres helse: funn fra undersøkelsen Levekår blant innvandrere 2005/2006 [Social differences in the health of immigrants based on data from the Survey on Living Conditions among Immigrants 2005/2006].

[27] Bråthen M. Levekår på vandring: velstand og marginalisering i Oslo [Living conditions in change: prosperity and marginalisation in Oslo], vol. 2007/05. Oslo: Forskningsstiftelsen FAFO [FAFO Institute]; 2007.

[28] Forland F (2009). Migrasjon og helse: utfordringer og utviklingstrekk [Migration and health: challenges and development].

[29] Pascoe EA, Smart RL (2009). Perceived discrimination and health: a meta-analytic review. Psychol Bull.

[30] Callen JL, Blundell L, Prgomet M (2008). Emergency department use in a rural Australian setting: are the factors prompting attendance appropriate?. Aust Health Rev.

[31] Alyasin A, Douglas C (2014). Reasons for non-urgent presentations to the emergency department in Saudi Arabia. Int Emerg Nurs.

[32] Buja A, Fusco M, Furlan P, Bertoncello C, Baldovin T, Casale P (2014). Characteristics, processes, management and outcome of accesses to accident and emergency departments by citizenship. Int J Public Health.

