# Supplementary material for: Is it a matter of urgency? A survey of assessments by walk-in patients and doctors of the urgency level of their encounters at a general emergency outpatient clinic in Oslo, Norway
Source: BMC Emerg Med. 2016 Jul 4;16:22. doi: 10.1186/s12873-016-0086-1 (PMC4932670; doi:10.1186/s12873-016-0086-1)
Supplement: Additional file 2: — Concordance for urgency assessment levels between patients and doctors stratified by consultation results. Additional table showing the concordance for urgency assessment levels between patients and doctors stratified by consultation results. A: Received final treatment at the emergency clinic, B: Admitted to hospital/decision unit or referred to specialist and C: RGP follow up. [file 12873_2016_86_MOESM2_ESM.pdf]

Additional file 2

Concordance for urgency assessment levels between patients and doctors stratified by consultation results. A: Received final treatment at the emergency clinic, B: Admitted to hospital/decision unit or referred to a specialist and C: RGP follow up

A:

	Doctors' assessment of urgency level			
	Less than one hour	Within a few hours	Non-urgent	Total
**Patients' assessment of urgency level**	n	n	n	n (%)
Less than one hour	4	76	158	238 (24)
Within a few hours	4	127	355	486 (49)
Non-urgent	0	43	232	275 (27)
Total n (%)	8 (1)	246 (24)	745 (75)	999 (100)

Concordance: Kendall tau-b score = 0.143 ($p < 0.001$)

B:

	Doctors' assessment of urgency level			
	Less than one hour	Within a few hours	Non-urgent	Total
**Patients' assessment of urgency level**	n	n	n	n (%)
Less than one hour	7	73	14	94 (39)
Within a few hours	8	99	15	122 (50)
Non-urgent	0	15	13	28 (11)
Total n (%)	15 (6)	187 (77)	42 (17)	244 (100)

Concordance: Kendall tau-b score = 0.145 ($p = 0.029$)

C:

	Doctors' assessment of urgency level			
	Less than one hour	Within a few hours	Non-urgent	Total
**Patients' assessment of urgency level**	n	n	n	n (%)
Less than one hour	1	14	31	46 (26)
Within a few hours	0	19	74	93 (52)
Non-urgent	0	4	36	40 (22)
Total n (%)	1 (1)	37 (20)	141 (79)	179 (100)

Concordance: Kendall tau-b score = 0.185 ($p = 0.008$)
