# Supplementary material for: Is it a matter of urgency? A survey of assessments by walk-in patients and doctors of the urgency level of their encounters at a general emergency outpatient clinic in Oslo, Norway
Source: BMC Emerg Med. 2016 Jul 4;16:22. doi: 10.1186/s12873-016-0086-1 (PMC4932670; doi:10.1186/s12873-016-0086-1)
Supplement: Additional file 3: — Logistic regression analysis for patients’ and doctors’ assessment of urgency level. Additional table presenting a logistic regression model for patients’ and doctors’ assessment of urgency level (dependent variable: ‘immediate’ versus ‘non-urgent’) using occupational status as a proxy for socioeconomic status. (PDF 181 kb) [file 12873_2016_86_MOESM3_ESM.pdf]

Additional file 3

	Patients' assessment		Doctors' assessment	
	**Unadjusted**	**Adjusted**	**Unadjusted**	**Adjusted**
	OR (95% CI)	OR (95% CI)	OR (95% CI)	OR (95% CI)
**Country/region of origin**				
Norway	1	1	1	1
Nordic countries	0.66 (0.45-0.96)*	0.86 (0.52-1.41)	0.64 (0.42-0.97)*	0.73 (0.45-1.20)
West Eur. / N. America and Oceania	1.18 (0.62-2.25)	1.00 (0.47-2.13)	0.92 (0.47-1.81)	1.10 (0.54-2.24)
East Europe	3.18 (1.76-5.74)**	2.62 (1.36-5.02)*	0.76 (0.49-1.16)	0.82 (0.51-1.33)
Asia with Turkey	3.68 (2.34-5.77)**	3.76 (2.25-6.28)**	1.04 (0.77-1.41)	0.99 (0.71-1.39)
Africa	4.25 (2.42-7.47)**	9.26 (3.97-21.6)**	0.49 (0.33-0.72)**	0.63 (0.41-0.97)*
Latin America	1.70 (0.64-4.55)	1.52 (0.52-4.81)	1.21 (0.53-2.80)	1.16 (0.47-2.82)
**Gender**				
Female		1		1
Male		1.16 (0.89-1.52)		0.95 (0.75-1.18)
**Age**				
0 – 19		1		1
20 – 39		0.94 (0.69-1.28)		1.25 (0.94-1.65)
40 – 59		2.00 (1.23-3.26)*		1.99 (1.39-2.85)**
≥ 60		1.83 (0.76-4.43)		2.01 (1.06-3.80)*
**Occupational status**				
Employee		1		1
Social welfare benefits[1]		1.33 (0.73-2.44)		0.92 (0.59-1.42)
Unemployed		0.92 (0.46-1.87)		1.19 (0.68-2.07)
Homemaker		2.50 (0.56-11.1)		1.03 (0.50-2.13)
Pensioner		1.49 (0.51-4.36)		1.28 (0.62-2.64)
Student		0.61 (0.44-0.86)*		0.87 (0.64-1.20)
Other		0.73 (0.39-1.86)		0.75 (0.36-1.53)
**Self-reported RGP status**				
Registered		1		1
Not registered		0.73 (0.47-1.13)		0.95 (0.64-1.40)
**Time of consultation**				
08:00am – 03:59pm		1		1
04:00pm – 10:59pm		1.10 (0.84-1.44)		1.17 (0.92-1.47)
11:00pm – 07:59am		2.79 (1.53-5.06)**		2.32 (1.58-3.42)**

[1]On sick leave/disability benefit/rehabilitation benefit. Def. RGP: regular general practitioner
Norwegians used as the reference group. OR (Odds ratio).
*Significant result at the p < 0.05 level, ** p < 0.001
