# Supplementary figures and images for: Is it a matter of urgency? A survey of assessments by walk-in patients and doctors of the urgency level of their encounters at a general emergency outpatient clinic in Oslo, Norway
Source: BMC Emerg Med. 2016 Jul 4;16:22. doi: 10.1186/s12873-016-0086-1 (PMC4932670; doi:10.1186/s12873-016-0086-1)

Additional file 4

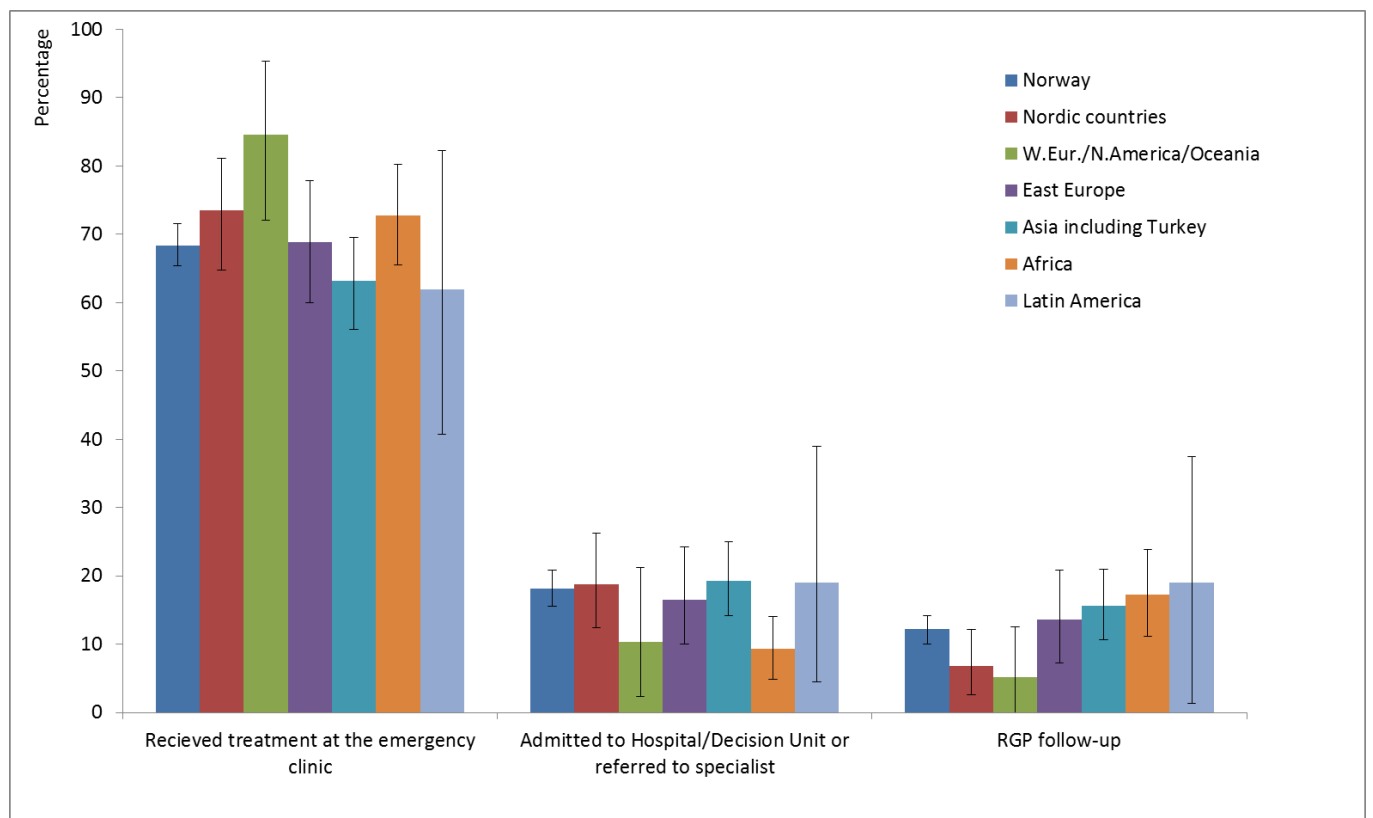

Supplement: Additional file 4: — Consultation results for Norwegians and immigrants based on region of origin. Additional table showing the consultation results according to the patients’ region of origin. (PDF 117 kb) [file 12873_2016_86_MOESM4_ESM.pdf]
